# Clinical impact of thyroglobulin (Tg) and Tg autoantibody (TgAb) measurements in needle washouts of neck lymph node biopsies in the management of patients with papillary thyroid carcinoma

**DOI:** 10.1590/2359-3997000000241

**Published:** 2017-01-27

**Authors:** M. Cecilia Martins-Costa, Rui M. B. Maciel, Teresa S. Kasamatsu, Claudia C. D. Nakabashi, Cleber P. Camacho, Felipe Crispim, Elza S. Ikejiri, M. Conceição O. Mamone, Danielle M. Andreoni, Rosa Paula M. Biscolla

**Affiliations:** 1 Departamento de Medicina Escola Paulista de Medicina Universidade Federal de São Paulo São Paulo SP Brasil Centro de Doenças da Tireoide e Laboratório de Endocrinologia Molecular e Translacional, Divisão de Endocrinologia, Departamento de Medicina, Escola Paulista de Medicina da Universidade Federal de São Paulo (EPM- -Unifesp), São Paulo, SP, Brasil; 2 Departamento de Medicina Universidade de Fortaleza Fortaleza CE Brasil Departamento de Medicina, Universidade de Fortaleza (Unifor), Fortaleza, CE, Brasil; 3 Centro de Doenças da Tireoide Instituto Israelita de Ensino e Pesquisa Albert Einstein São Paulo SP Brasil Centro de Doenças da Tireoide, Instituto Israelita de Ensino e Pesquisa Albert Einstein (IIEPAE), São Paulo, SP, Brasil; 4 São Paulo SP Brasil Fleury Medicina e Saúde, São Paulo, SP, Brasil

**Keywords:** Fine needle aspiration biopsy, anti-thyroglobulin antibody, thyroglobulin, papillary thyroid cancer, neck lymph nodes

## Abstract

**Objectives:**

The presence of thyroglobulin (Tg) in needle washouts of fine needle aspiration biopsy (Tg-FNAB) in neck lymph nodes (LNs) suspected of metastasis has become a cornerstone in the follow-up of patients with papillary thyroid carcinoma (PTC). However, there are limited data regarding the measurement of anti-Tg antibodies in these washouts (TgAb-FNAB), and it is not clear whether these antibodies interfere with the assessment of Tg-FNAB or whether there are other factors that would more consistently justify the finding of low Tg-FNAB in metastatic LNs.

**Materials and methods:**

We investigated 232 FNAB samples obtained from suspicious neck LNs of 144 PTC patients. These samples were divided according to the patient’s serum TgAb status: sTgAb- (n = 203 samples) and sTgAb+ (n = 29). The TgAb-FNAB levels were measured using two different assays. Tg-FNAB was also measured using two assays when low levels (< 10 ng/mL) were identified in the first assay of the metastatic LNs from the sTgAb+ samples.

**Results:**

The TgAb-FNAB results were negative in both assays in all samples. Low levels of Tg-FNAB were identified in 11/16 of the metastatic LNs of the sTgAb+ patients and 16/63 of the sTgAb- patients (p < 0.05) using assay 1. The measurement of the Tg-FNAB levels using assay 2 indicated additional metastases in 5 LNs of the sTgAb+ patients.

**Conclusions:**

Factors other than the presence of TgAb-FNAB may contribute to the higher number of metastatic LNs with undetectable Tg-FNAB in the sTgAb+ group. In addition, the measurement of Tg-FNAB using different assays was useful to enhance the diagnosis of metastatic LNs, particularly when cytological and Tg-FNAB results are discordant.

## INTRODUCTION

Papillary thyroid carcinoma (PTC) is the most common endocrine malignancy, with a propensity for cervical lymphatic spread, which occurs in 20% to 50% of patients based on reports of surgical pathological specimens using standard methods of description; however, the prevalence may reach 90% of patients when the surgical sample is scrutinized in detail for micrometastases (1,2).

The confirmation of malignancy in lymph nodes (LNs) with a suspicious ultrasonographic (US) appearance is achieved by US-guided fine needle aspiration biopsy (FNAB) for cytological study (cyto-FNAB) and the measurement of thyroglobulin levels (Tg) in needle washouts (Tg-FNAB) (3-8).

Considering only the data from cyto-FNAB, approximately 6% to 8% of metastases are misdiagnosed as a result of false-negative results (9,10). These results may occur in cases of small or cystic LN metastases, which reflect a lack of tumor cells detected during the FNAB procedure (11). Nevertheless, the use of Tg-FNAB alone has several limitations, and despite being well established as an important tool in the investigation of suspicious LN metastases, Tg-FNAB presents variable results between metastatic and non-metastatic LNs (3,4,11-23). This subject has been the topic of recent reviews and meta-analysis studies (12,13).

Another aspect for consideration during the follow-up of patients with PTC is the interpretation of serum Tg (sTg) values in the presence of serum anti-Tg antibodies (sTgAb), which occurs in approximately 25% of patients with PTC; these antibodies may interfere with the measurement of sTg, which compromises the use of this tumor marker in the follow-up of patients with PTC (24). It remains controversial whether these antibodies also interfere with the assessment of TgAb-FNAB. Previous studies on this topic have included a limited number of patients with positive serum anti-Tg antibodies (sTgAb+) (25-27) and limited direct measurements of TgAb-FNAB (16,18,20,25,26).

Therefore, the objectives of the present study were to: 1) assess the presence of TgAb in a substantial amount of washout fluid samples (TgAb-FNAB) of cervical LNs suspicious for PTC metastases; 2) analyze whether the presence of serum sTgAb+ interfered with the assessment of Tg-FNAB for the management of neck lymphadenopathy in PTC patients; and 3) compare the TgAb values of the serum and FNAB washout.

## MATERIALS AND METHODS

### Patients

We assessed 144 patients who presented suspicious LNs via US assessment during follow-up for PTC. These patients were followed by a single team of physicians at associated Thyroid Disease Centers at the Division of Endocrinology, Department of Medicine, *Escola Paulista de Medicina, Universidade Federal de Sao Paulo* and the *Instituto Israelita de Ensino e Pesquisa Albert Einstein*, both in Sao Paulo, Brazil.

Suspicious LNs were submitted to FNAB guided by neck US assessment. The minimum follow-up period was 4 years after the last FNAB. Some patients had more than one FNAB performed when multiple suspicious LNs appeared during the follow-up or the FNAB had to be repeated because of inadequate or non-diagnostic cyto-FNAB results with undetectable Tg-FNAB values. Two hundred eighty-six FNABs were performed, and 232 FNAB samples were analyzed. Fifty-four FNAB samples were excluded because the patients were lost during follow-up (n = 32) or the diagnosis of lymphadenopathy could not be confirmed (n = 22), as cytopathology indicated that these samples were obtained from other cervical lesions rather than LNs.

The confirmation of LN metastases was obtained via histopathological examination after surgery or the ^131^I uptake after radioiodine treatment. In two particular situations, LNs that initially fulfilled suspicious criteria via US assessment were considered reactive over time: 1) LNs that spontaneously disappeared at the subsequent US assessment performed during follow-up; and 2) LNs that initially had inadequate or non-diagnostic cytology results on the first FNAB attempt and presented a reactive cytology with Tg-FNAB < 10 ng/mL after a second FNAB.

All diagnostic procedures were performed in accordance with the regulations of the local ethics committee. Written informed consent was obtained from each patient.

### Assays

Tg-FNAB was measured with a commercial immunofluorometric assay using monoclonal antibodies (DELFIA^®^, PerkinElmer, Turku, Finland), with a functional sensitivity of 1.0 ng/mL (assay 1). A second immunofluorometric Tg assay (assay 2), using mono and polyclonal antibodies, was performed on the washout fluids of sTgAb+ patients with a Tg-FNAB value < 10 ng/mL in assay 1 to determine whether low Tg-FNAB persisted using a different method. Assay 2 has a functional sensitivity of 0.3 ng/mL (28). The Tg assay 2 was assessed only in this specific group of patients for whom the TgAb-FNAB results, in the case of positivity by contamination with sTgAb, could interfere with the Tg-FNAB levels. We verified whether these lower Tg-FNAB levels would be maintained when examined using another assay.

The Tg-FNAB cutoff point for the diagnosis of malignancy has not been unanimously established in the literature (12,13). We used the value of 10 ng/mL, which is consistent with the majority of published reports (11,14,20-22).

TgAb was measured using an in house immunofluorometric assay in both serum and FNAB washout samples, and negative values for TgAb were considered < 40 IU/mL (29). In addition, we also measured TgAb in FNAB washout samples using a chemiluminescence immunoassay (ECLIA Roche, Mannheim, Germany). The cutoff value for positive TgAb was 115 IU/mL.

### Ultrasonography

A cervical US assessment was performed using a linear, multi-frequency, 7.5-10-MHz transducer and integrated using color-Doppler examination by the same radiologist. The images were obtained in transversal and longitudinal planes by scanning the hyperextended neck, which enabled the visualization of the central compartment. All cervical LNs were identified, localized, and measured. LNs with 1 or more suspicious features indicative of PTC neck metastasis, i.e., round shape, hypoechogenic aspect, absence of hilum, non-homogeneous pattern, including fluid areas, and intralesional punctate calcifications, were submitted to FNAB.

### Cyto-FNAB and Tg-FNAB

US-guided FNAB was performed using a 22-25-gauge needle attached to a 10-mL syringe inserted into the LN under US visual control. The needle was repeatedly moved inside each LN until the needle hub was filled with material. The smears (4-8 per LN) were immediately fixed and stained with panoptic dye. All cytological examinations were performed by the same cytopathologist.

The cyto-FNAB results were classified into 3 distinct diagnostic categories: 1) reactive: presence of lymphocytes and occasional plasma cells without malignant epithelial cells; 2) inadequate or non-diagnostic: presence of blood cells and absence of inflammatory and epithelial cells; and 3) positive for PTC metastases: presence of epithelial cells with malignant cytological characteristics (*e.g*., abnormal nuclear shape, nuclear enlargement and nuclear polymorphisms, presence of papillae, and/or characteristic nuclear changes, such as grooves and pseudo-inclusions).

Following the smear preparation, the needle was washed with 1 mL of saline, and the solution was processed for Tg-FNAB measurement and subsequently stored at -20°C. The same samples were used to measure the TgAb-FNAB levels.

### Statistical analysis

The number of metastatic LNs in Group 1 (sTgAb-) and Group 2 (sTgAb+), as well as their Tg-FNAB levels (assay 1) and cyto-FNAB results were compared using Fisher’s Exact Tests and χ^2^ tests as appropriate, with a two-tailed p < 0.05 considered statistically significant. The sensitivity, specificity, positive predictive value and negative predictive value of a positive Tg-FNAB were calculated using assay 1 (Tg-FNAB ≥ 10 ng/mL). All analyses were performed using the Statistical Package for Social Science professional software version 15.0 (SPSS, Chicago, IL, USA).

## RESULTS

**Figure 1:**
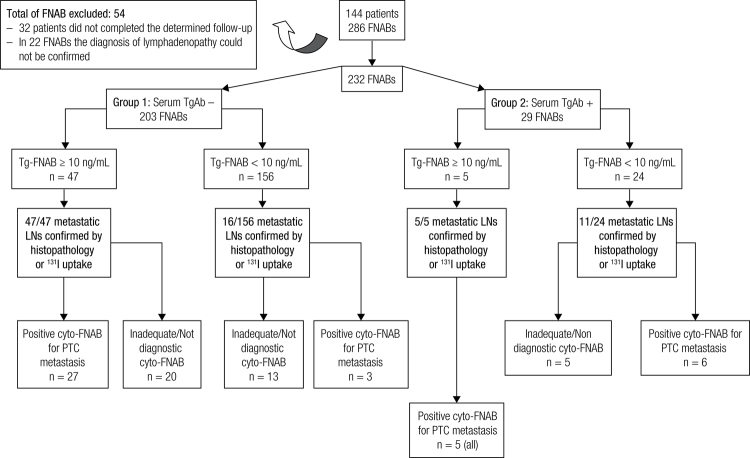
General overview of the study with detailing of Tg-FNAB (by assay 1-(immunofluorometric, with monoclonal antibodies) and cyto-FNAB results of Group 1 (sTgAb–) and Group 2 (sTgAb+).

A general overview of the present study that indicates the results of 232 FNABs in 144 patients is presented in Figure 1. The patients were divided into 2 groups: Group 1, which included patients who presented negative sTgAb values (sTgAb-) (203 FNABs), and Group 2, which included patients who presented positive sTgAb values (sTgAb+) (29 FNABs). These groups were subsequently subdivided according to the Tg-FNAB levels (Tg-FNAB ≥ 10 ng/mL and Tg-FNAB < 10 ng/mL) and the cyto-FNAB results (reactive, inadequate/non-diagnostic or positive for PTC metastasis) (Figure 1).

We identified metastases in 63 (31%) LNs in Group 1 (sTgAb-) and 16 (55%) LNs in Group 2 (sTgAb+). This difference was statistically significant (p < 0.05, Fisher’s Exact test) (Table 1). All metastatic LNs presented classical and follicular PTC histological variants.

**Table 1:** Comparison of Tg-FNAB levels (assay 1) and cyto-FNAB results of metastatic lymph nodes from Group 1 (sTgAb-) and Group 2 (sTgAb+)

	GROUP 1 sTgAb-Metastatic/TOTAL (%) 63 / 203 (31%)^a^		GROUP 2 sTgAb+Metastatic/TOTAL (%) 16 / 29 (55%)^a^	
	Tg-FNAB < 10 ng/mL	Tg-FNAB ≥ 10 ng/mL	Tg-FNAB < 10 ng/mL	Tg-FNAB ≥ 10 ng/mL
	n	n	n	n
Inadequate/not diagnostic cyto-FNAB	13	20	5	0
Positive for metastasis cyto-FNAB	3	27	6	5
TOTAL	16 (25.4%)^b^	47 (74.6%)^c^	11 (68.8%)^b^	5 (31.3%)^c^

^a^ Statistical difference between groups (p < 0.05) using Fisher’s Exact Test 2 test.

^b,c^ Statistical difference between groups (p < 0.05) using χ^2^ test.

Sixteen of the 63 metastatic LNs in Group 1 and 11 of the 16 metastatic LNs in Group 2 had Tg-FNAB levels < 10 ng/dL. Therefore, Group 2 (sTgAb+) had a higher number of metastatic LNs with Tg-FNAB values < 10 ng/dL than Group 1 (68.8% in Group 2 vs. 25.4% in Group 1; p < 0.05, χ^2^ test) (Table 1). Consistent with the serum results, this finding suggests that the presence of sTgAb may underestimate the measurement of Tg-FNAB.

The cutoff of Tg-FNAB of 10 ng/mL (assay 1) for the diagnosis of metastatic PTC in cervical LNs in Group 1 (sTgAb-) exhibited a sensitivity of 74.6%, with a specificity of 100%, a positive predictive value of 100% and a negative predictive value of 89.7%; Group 2 (sTgAb+) exhibited a sensitivity of 31.2%, with a specificity of 100%, a positive predictive value of 100% and a negative predictive value of 54.2%. Notably, it was not possible to perform the same analysis for Tg-FNAB using assay 2 because it was only tested in the washout fluids of the sTgAb+ patients with Tg-FNAB levels < 10 ng/mL in assay 1.

To evaluate the potential differences in the performance of Tg assay 1 in the sTgAb+ patients (Group 2), we performed a different Tg-FNAB assay (using mono and polyclonal antibodies, Tg assay 2) in all washout fluid samples of metastatic LNs with Tg-FNAB levels < 10 ng/mL using assay 1 (using monoclonal antibodies). We obtained different results between both assays in 4 patients whose cyto-FNABs were positive for PTC metastases and the levels of Tg-FNAB were low (< 10 ng/mL) according to assay 1 and high (≥ 10 ng/mL) according to assay 2 (Table 2). In these patients, the measurements of Tg-FNAB using assay 2 were consistent with the cyto-FNAB measurements. These findings also indicated that the presence of sTgAb could interfere with the interpretation of Tg-FNAB measurements. Furthermore, these findings suggest methods that employ polyclonal antibodies, such as radioimmunoassays and assay 2, appeared more resistant to TgAb interference (24). Moreover, in one patient, a suspicious LN based on US assessment presented inadequate/non-diagnostic cyto-FNAB, and the level of Tg-FNAB was 8.7 ng/mL according to assay 1 and 142 ng/mL according to assay 2. This patient underwent ^131^I treatment, and the subsequent scintigraphy indicated substantial ^131^I uptake in the area of the corresponding LN. Consequently, in this patient, the measurement of Tg-FNAB using assay 2 was important for the diagnosis of metastasis.

**Table 2:** Comparison of Tg-FNAB by assay 1 (immunofluorometric, with monoclonal antibodies) and assay 2 (immunofluorometric with mono and polyclonal antibodies) and cytological results of metastatic lymph nodes (LNs) of Group 1 (sTgAb-) and Group 2 (sTgAb+) with Tg-FNAB levels < 10 ng/mL by assay 1

	Tg-FNAB Assay 1	Tg-FNAB Assay 2
Reactive cyto-FNAB	–	–
Inadequate/not diagnostic cyto-FNAB	8.7	142
	< 1.0	1.6
	< 1.0	0.5
	< 1.0	0.9
	< 1.0	0.6
Positive for metastasis cyto-FNAB	< 1.0	28.6
	5.9	42.7
	< 1.0	142
	< 1.0	1.4
	2.1	101
	< 1.0	1.1

However, the direct measurement of TgAb-FNAB based on immunofluorometric assays was negative in all 232 samples of both groups, including the FNAB washouts of the sTgAb+ patients. Using a second assay (electrochemiluminescence assay, ECLIA, Roche), we identified 7 samples with positive TgAb-FNAB values, all of which were obtained from Group 1 (sTgAb-) patients, with high levels of Tg-FNAB, which likely lead to false elevations of TgAb-FNAB. Moreover, this artifact represents an established competitive effect that occurs when the Tg levels are > 2000 ng/mL, and these values must be considered false-positive results, as described by the manufacturer.

## DISCUSSION

The evaluation of cervical LNs is difficult during the follow-up of PTC patients because inflammatory LNs are frequently present. The use of Tg-FNAB is well recognized as a valuable instrument in the examination of suspicious LN metastases (3,4,11-23). However, there are limited data regarding factors that may interfere with the diagnostic sensitivity of Tg-FNAB, such as the presence of TgAb in washout fluid samples.

The presence of TgAb in FNAB washout fluids may reflect the active LN synthesis of TgAb (30) or the contamination of the washout fluids with blood; thus, we measured the TgAb-FNAB levels in both positive and negative sTgAb patients (n = 232). The results indicated that all washout fluid samples obtained from the patients in both groups were negative for TgAb-FNAB based on immunofluorometric assays. Notably, when we retested the TgAb-FNAB levels using a chemiluminescence immunoassay, we identified 7 samples in Group 1 (sTgAb-) patients with positive TgAb-FNAB values. However, as previously described, these patients had extremely high levels of Tg-FNAB, and according to the manufacturer’s description, these results must be interpreted as false-positive because of the high titers of Tg-FNAB. We thus considered that all washout fluid samples of Groups 1 and 2 were negative for TgAb-FNAB in both assays performed. However, we raised several speculations to justify the negative results of TgAb-FNAB in all samples in the present study, including the samples obtained from sTgAb+ patients: 1) active LN synthesis of TgAb, which may eventually occur, was scarce in the samples, and consequently, there was no detection of TgAb in the washouts; 2) there was no contamination of the washout fluid with blood during the FNAB procedure in the sTgAb+ patients; and 3) the washouts of LNs perfused with blood that contain TgAb may have a low concentration of these antibodies; however, it is insufficient to measure.

Other studies have presented similar results. Following the inclusion of a limited number of TgAb-FNAB measurements of LN washout in sTgAb+ patients, the authors did not detect TgAb via direct measurement in the washout fluids of LNs, which suggests that sTgAb do not interfere with the detection of Tg-FNAB levels (16,18,20,26).

Boi and cols. evaluated washout fluid samples from 8 sTgAb+ patients and suggested that the presence of TgAb-FNAB may interfere with the interpretation of Tg-FNAB results because lower levels of Tg-FNAB were detected in 2 metastatic LNs with positive TgAb-FNAB levels compared with metastatic LNs with negative TgAb-FNAB levels (25). These authors proposed that this interference is minimized by the high Tg concentrations in the FNAB washouts of metastatic LNs, which saturate TgAb binding sites and therefore do not compromise the value of Tg-FNAB in the diagnosis of metastases. The results of the present study, which included a larger number of patients (11/24 patients sTgAb+ and Tg-FNAB < 10 ng/mL, Group 2, Figure 1), confirm this finding.

TgAb-FNAB was negative in all samples examined in the present study; thus, further comparisons between the TgAb values in the serum and FNAB washout would not be plausible. Instead, we considered other potential reasons why low levels of Tg-FNAB were present in the metastatic LNs of patients with PTC, including difficulties in sampling a representative area of neoplasia during the FNAB procedure, heterogenic Tg production by metastatic cells and differences in Tg assay performance.

Regarding the difficulties in sampling a representative area of neoplasia during FNAB, we identified metastatic LNs with Tg-FNAB < 10 ng/mL in both Group 1 (n = 16) and Group 2 (n = 11). We proposed that this Tg-FNAB value represented a true negative result in most cases based on the finding that the majority of these LNs also exhibited inadequate/non-diagnostic cyto-FNAB results (Group 1: n = 13/16 and Group 2: n = 5/11) (Table 1). However, this result may also reflect the non-homogeneous patterns and fluid areas that occur in some metastatic LNs.

In the present study, we did not identify undifferentiated variants of PTC carcinoma in metastatic LNs with low Tg-FNAB levels (< 10 ng/mL). In both Groups 1 and 2, all metastatic LNs with low Tg-FNAB values presented classical and follicular PTC histological variants. However, variable levels of Tg-FNAB have been described, even in samples with the same variant, which reflects heterogenic Tg production by differentiated metastatic cells (24).

Differences in Tg assays were also identified in the present study. The finding of negative TgAb-FNAB in all samples and a higher percentage of metastatic LNs with Tg-FNAB < 10 ng/dL in the sTgAb+ patients than the sTgAb- patients (68.75% vs. 25.4%, respectively, p < 0.05) prompted us to perform a second Tg assay in the washout fluids of all proven metastatic LNs of the sTgAb+ patients with Tg-FNAB < 10 ng/dL (Table 2). In some patients, we obtained different results between both assays. The Tg-FNAB levels according to assay 2 were consistent with the cytological results in 4 LNs with positive cyto-FNAB for PTC metastasis with undetectable Tg-FNAB based on assay 1, and these LNs were metastatic. The Tg-FNAB level of 142 ng/mL detected using assay 2 also clarified the diagnosis of a metastatic LN with inadequate/non-diagnostic cyto-FNAB and a Tg-FNAB level of 8.7 ng/mL according to assay 1 (Table 2).

Similar to the results obtained in the present study, Jeon and cols. (27) and Jo and cols. (31) also identified a higher percentage of LNs with low levels of Tg-FNAB in sTgAb+ patients than sTgAb- patients. These authors concluded that the presence of sTgAb interfered with the Tg-FNAB levels; however, these researchers did not measure TgAb in FNAB washout fluid samples (27,31).

In conclusion, we propose that other factors, rather than the presence of TgAb, may contribute to the higher number of metastatic LNs with undetectable Tg-FNAB identified in the sTgAb+ group because TgAb-FNAB was negative in all samples according to 2 different assays. Furthermore, for the analysis of Tg-FNAB in the sTgAb+ patients, assay 2 (mono/polyclonal) had a better performance in the detection of metastatic LNs than the monoclonal assay; however, it exhibited limitations in the identification of some LN metastases. Nevertheless, this analysis was useful in some cases in which there was no concordance between the cyto-FNAB and Tg-FNAB results or when the cyto-FNAB was inadequate/non-diagnostic and the Tg-FNAB levels were low based on the monoclonal assay. Therefore, we proposed that the measurement of Tg using another assay would be helpful in LNs with a positive cytology for PTC and low levels of Tg-FNAB using the first assay and in cases of inadequate/non-diagnostic cytology and low Tg-FNAB levels. However, in this latter situation, we emphasize that the low value of Tg-FNAB may persist in every additional assay assessed, as a matter of material scarcity. When employed alone, cyto-FNAB or Tg-FNAB (independent of the tested assay) did not provide a guarantee of establishing the diagnosis of cervical lymphadenopathy in patients.

In addition, false-positive and false-negative results may occur, which reinforce the recommendations that these LNs require monitoring and physicians must interpret clinical, laboratory, cytology, and ultrasound data together to achieve successful management in the follow-up of patients with PTC.
